# The impact of care pathways for patients with proximal femur fracture: rationale and design of a cluster-randomized controlled trial

**DOI:** 10.1186/1472-6963-12-124

**Published:** 2012-05-24

**Authors:** Kris Vanhaecht, Walter Sermeus, Jan Peers, Cathy Lodewijckx, Svin Deneckere, Fabrizio Leigheb, Steven Boonen, An Sermon, Paulo Boto, Rita Veloso Mendes, Massimiliano Panella

**Affiliations:** 1 Health Services Research Group, School of Public Health, KU Leuven, University of Leuven, Kapucijnenvoer 35, 3000, Leuven, Belgium; 2 European Pathway Association, Kapucijnenvoer 35, 3000, Leuven, Belgium; 3 Western Norway Research Group on Integrated Care, Helse Fonna, 5504, Haugesund, Norway; 4 Department of Pulmonology, University Hospitals Leuven, Herestraat 49, 3000, Leuven, Belgium; 5 Department of Clinical and Experimental Medicine, Amedeo Avogadro University of Eastern Piedmont, Via Duomo, 13100, Vercelli, Italy; 6 Department of Geriatrics and Leuven University Center for Metabolic Bone Diseases, University Hospitals Leuven, KU Leuven, University of Leuven, Herestraat 49, 3000, Leuven, Belgium; 7 Department of Orthopedics & Traumatology, University Hospitals Leuven, Herestraat 49, 3000, Leuven, Belgium; 8 National School of Public Health, Avenido Padre Cruz 1600-560, Lisbon, Portugal

## Abstract

**Background:**

Proximal femur fracture (PFF) is associated with considerable morbidity and mortality. The European Quality of Care Pathway (EQCP) study on PFF (NCT00962910) was designed to determine how care pathways (CP) for hospital treatment of PFF affect consistency of care, adherence to evidence-based key interventions, and clinical outcome.

**Methods/Design:**

An international cluster-randomized controlled trial (cRCT) will be performed in Belgium, Ireland, Italy and Portugal. Based on power analyses, a sample of 44 hospital teams and 437 patients per arm will be included in the study. In the control arm, usual care will be provided. Experimental teams will implement a care pathway which will include three active components: a formative evaluation of quality and organization of the care setting, a set of evidence-based key interventions, and support of the development and implementation of the CP. Main outcome will be the six-month mortality rate.

**Discussion:**

The EQCP study constitutes the first international cRCT on care pathways. The EQCP project was designed as both a research and a quality improvement project and will provide a real-world framework for process evaluation to improve our understanding of why and when CP can really work.

**Trial registration number:**

NCT00962910

## Background

Fracture of the proximal femur constitutes one of the most devastating complications of osteoporosis. Within the European Union, more than 400,000 women and 100,000 men sustain a hip fracture every year [1]. The worldwide annual number of hip fractures amounts to about 1,800,000. Because the number of elderly people is rising, a continued increase in incidence of fractures is expected [1]. People with a proximal femur fracture experience a clinically important decline in functional status with considerable loss in quality of life [2,3]. Within one year after sustaining a hip fracture, close to 20% of individuals will have to be institutionalized because of the fracture and because of its functional consequences. Overall, hip fractures represent one of the main causes of hospitalization, institutionalization, and mortality in old age [4]. According to a meta-analysis published in 2010, older adults have a 5- to 8-fold increase in their risk of all-cause mortality during the first 3 months after hip fracture (3). This excess mortality persists over time, even 10 years after sustaining the fracture; both women and men are affected, although, at any given age, excess annual mortality after hip fracture is higher in men than in women [3].

Organizing and standardizing the care process for these patients, with a focus on quality, efficiency, and accessibility should be one of the priorities over the next few years for clinicians, healthcare managers, and policy makers. One of the methods to (re)organize a care process is the development and implementation of a care pathway. Care pathways, also known as clinical pathways or critical pathways, are used worldwide for a variety of patient groups [5-13]. A care pathway is defined as a complex intervention for mutual decision making and organization of predictable care for a well-defined group of patients during a well-defined period. Defining characteristics of pathways include: explicitly stating the goals and key elements of care based on evidence, best practice and patient expectations; facilitating communication and coordination of roles and sequencing the activities of the interprofessional care team; optimizing communication with patients and their relatives; documenting, monitoring, and evaluating variances and outcomes; and, finally, identifying relevant resources [12,14-16].

A care pathway is explicitly defined as a “complex intervention” [12,17-19]. Complex interventions, also known as multi-component interventions, have been built from a number of components that may act both independently and interdependently [20,21]. Although they may be difficult to specify, these interacting components seem essential for the proper functioning of the intervention. Considering a spectrum of low to high complexity, developing a drug would be at the low end of the spectrum while assessing the effect of a stroke unit would be at the high end. The more it is difficult to exactly define the “active components” of an intervention and how these interrelate, the more it is likely that the intervention is a complex one [19,20,22]. Care pathways seem to be at the higher end of the complexity spectrum. Typical active ingredients of a care pathway include the promotion of interdisciplinary teamwork, the integration of a package of evidence-based key interventions, and the active follow-up of care processes [6,12,13,23,24].

A recent Cochrane review concluded that care pathways result in reduced in-hospital complications and improved documentation, without negatively impacting length of stay or hospital costs [25]. However, these effects may vary widely and may not always meet expectations. To gain insight into the active components of complex care pathways, one needs to evaluate the context of the interventions and the mechanisms involved [14,20,21,26,27]. Multicenter trials that include these evaluations are critical to fully understand how and when care pathways are effective [12,28,29].

A literature search identified six reviews on the effect of pathways in patient groups that included hip fracture patients [25,30-34]. Because various types of patients were included in the reviews by Rotter et al. (2008, 2010), it was not possible to address any effect on hip fracture patients specifically [25,30]. A more patient-specific meta-analysis by Neuman et al. (2009) was limited by the lack of a common definition and concept on care pathway [31] and did not allow a formal comparison of the outcomes of the included studies. Moreover, in many of the primary studies included in the reviews, the components of the complex interventions were not always described [32-34]. Therefore, even when a care pathway was developed, many of the observed results could not be attributed directly to the pathway. Also, study designs were substantially different. Despite these limitations, the major conclusion that emerged from these reviews is that care pathways can significantly reduce the length of stay and have a positive impact on different outcomes. The results also suggest that mortality in hip fracture patients may not be the best parameter to assess quality of care as they may ignore important improvements in other outcomes that can be achieved by care pathways. Additional research is needed to evaluate the impact of care pathways on quality of care and clinical outcome in hip fracture patients.

To evaluate care pathway effectiveness, the European Pathway Association (E-P-A), an international not-for-profit association, launched the European Quality of Care Pathways (EQCP)-study on proximal femur fracture. Earlier, E-P-A launched a similar study on exacerbation of chronic obstructive pulmonary disease (COPD) [12,29].

### Objectives

The primary goal of the EQCP study on PFF is to evaluate care pathway effectiveness in the acute hospital setting. A secondary goal is to understand how and under what circumstances the implementation of a pathway for PFF is successful [29].

## Methods/Design

### The project

The European Quality of Care Pathways (EQCP) study is an international multicentre research project launched by the European Pathway Association (E-P-A) (http://www.E-P-A.org) [35]. The E-P-A is collaborating with the Center for Health Services and Nursing Research of the Faculty of Medicine of Leuven University (Belgium) and the School of Public Health of the Amedeo Avogadro University of Eastern Piedmont (Italy), which is taking the scientific lead in this study. The study will be executed in four countries: Belgium, Ireland, Italy and Portugal. In each country, a research centre is coordinating the project based on an internationally agreed protocol. In Belgium, the lead coordinating centre is the Center for Health Services and Nursing Research of the Faculty of Medicine of Leuven University. For Ireland, the lead centre is the Health Service Executive in Dublin. In Italy, the School of Public Health of the Amedeo Avogadro University of Eastern Piedmont is coordinating the project with support from ARESS Piemonte. In Portugal, the lead coordinating centre is the National School of Public Health in Lisbon. In each of the four countries, hospitals will be selected by E-P-A in close collaboration with a national coordinator. In each participating hospital, a study coordinator will be appointed to facilitate implementation of the care pathway. The study coordinator will be trained by the E-P-A team on how to develop and implement care pathways [12,29].

### Study design

**Figure 1:**
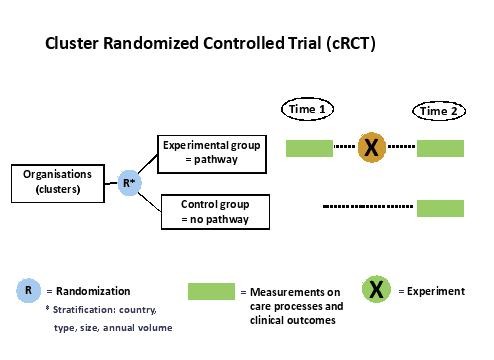
The EQCP study design.

To evaluate the effect of a care pathway, a cluster-randomized controlled trial (cRCT) will be conducted [12,17,18,20,22,36] (Figure 1). In cRCTs, organizations rather than individuals are randomized to an intervention and a control group, and outcomes are measured on an individual level within the clusters [20]. Each cluster consists of patients hospitalized for PFF in a particular hospital and cared for by a specific interprofessional care team.

Stratified randomization will be used to assign hospitals to an intervention group (development and implementation of an evidence-based care pathway) and a control group (no intervention/usual care). To ensure that clusters in both arms are in balance, clusters will be stratified according to country-level, hospital type (teaching versus non teaching), hospital size (<600 and ≥600 beds), and annual volume of patients (<300 and ≥300 patients). Hospitals included in the control group will have the opportunity to develop a care pathway one year later, based on the protocol and experience gained in the experimental group. Through this process, a network of high-performing organizations for PFF can be built.

### Inclusion – exclusion criteria

Organizations are included if the hospital management and the patient care team provide written consent to participate and, when randomized in the control group, agree not to develop and implement a pathway for PFF within the time frame of the study. All patients consecutively admitted for PFF will be considered for inclusion based on the following criteria: (i) provide written informed consent; (ii) minimum age of 65 years; (iii) closed fracture; (iv) eligible for surgical intervention; (v) American Society of Anesthesiology score 1, 2, or 3; and (vi) able to communicate in the native language. Each patient will be included only once in the study, at the initial admission, even if a patient is rehospitalized during the enrollment period. Exclusion criteria are: (i) severe dementia (based on DSM IV-criteria); pathological fracture; or a peri-prosthetic fracture.

### Study sample

Sample size calculation in a cRCT was based on the expected improvement in the main outcome variables [12,17,18,20,22]. The selection of main outcomes for the EQCP-study was based on three criteria: frequency of use in the literature, expert opinion and timing of the research project with respect to organizational constraints (sustainability of the design, time to include patients). Based on these criteria, six-month mortality rate was identified as the main study outcome for in-hospital management of PFF. Based on a power of 80% and an α of 0.05 (two-sided), it was calculated in Statistica® that 325 patients per arm are needed to observe a reduction from 15% to 8% [37,38]. After adjustment for the cluster design, based on two previous cRCTs by Panella et al. [17,18] (ICC: 0.018; IFF: 1.342; n = 20), the effective sample size increased to 437 patients per arm. Assuming a number of 20 consecutive admitted patients in each unit, 22 hospitals need to be included in each intervention and control group [17,18].

### The complex intervention: care pathway implementation in the intervention arm

**Figure 2:**
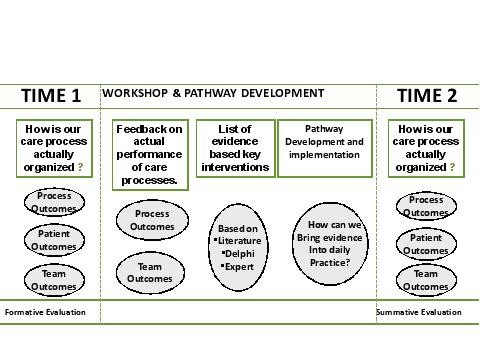
The EQCP complex intervention.

The care pathway will include three active components. (i) The *first* component is a formative evaluation of the quality and organization of the care process and will be performed by measuring performance on key interventions (see Figure 2) [12,39]. The formative evaluation is an evaluation of the actual performance of the care process in each of the experimental clusters. This evaluation is led by local contact persons who each followed a workshop. The goal of this workshop was to inform the contact persons and to standardize the procedure. During this one day workshop the national coordinator, supported by the international research team, presented how and what to evaluate. The main focus was the multidisciplinary teamwork and the performance of the key interventions. Key interventions are those that have a significant impact on patient outcomes. These are performed by all professional groups within the patient care team that treats PPF patients, i.e., orthopedic surgeons, geriatricians, nurses, physiotherapists, and social workers. Key interventions are performed in the domains of pre- peri-, and postoperative care and include patient assessment, appropriate medication, execution of essential laboratory tests and medical imaging, passive and active mobilization, pain management, discharge management and patient information. Feedback will be provided on the data obtained to help the teams understand their performance on process indicators as potential areas for improvement and redesign of the actual care process. To this end, a formal evaluation will be performed before developing the care pathway. During this evaluation, performance on a set of key-interventions will be measured in 20 consecutive patients (see ii). All data will be transferred to the research center for analysis. A feedback report will be provided to allow the teams to benchmark their performance compared to all other teams participating in the study. (ii) A set of evidence-based key interventions will be provided to the team. This set will be based on an extensive literature review, Map of Medicine® (http://www.mapofmedicine.com), and consensus among international clinical experts using a Delphi-survey. The key interventions and outcomes will include both in-hospital interventions and information for a safe discharge. In this second part of the intervention, each of the experimental teams will receive and discuss a feedback report. This feedback report will provide information on the actual performance (see i). Per team a local contact person and 2 representatives of the team are invited to a seminar. During this seminar the international coordinator, supported by the national coordinator will present the findings. The lead in this seminar is with the international coordinator to make sure the data are presented in a standardized way. (iii) All study coordinators will be trained to improve the organization of the care process by developing and implementing a care pathway, based on the evaluation of the care process and the set of evidence-based key interventions. In the training workshops, a care pathway implementation protocol will be used that is based on the Deming-PDSA cycle, a generally accepted standard method for quality improvement [40]. This workshop will be led by the international coordinator. He will be supported by local experts in care pathways who each followed a five day international summer school on the development, implementation and evaluation of care pathways [41]. This summer school was organized by the European Pathway Association [35].

During the intervention different meetings with the study coordinators and team member representatives will be organized to present and discuss the feedback report and to discuss actual bottlenecks in implementing the care pathway. Change will be promoted by exchanging best practices among participants. During these meetings, local clinical champions and team change experts will help and stimulate the study coordinators to effectively share knowledge [12,29].

Along with randomized controlled trials, in complex interventions, qualitative methods such as process evaluations are advised to promote quality care [42]. In this regard, the context in which the care pathway was developed and the implementation process itself will be evaluated using qualitative, observational approaches (e.g., semi-structured interviews with key stakeholders) during and after pathway-implementation.

### The control group

In the control group, the complex intervention will not be implemented and the teams will provide usual care. Control hospitals agree not to develop a care pathway during the study period.

### Measurements

To measure the effect of the care pathways, a set of process and outcome indicators for PFF were developed. The primary outcome measure is 6-month Mortality. Next to this indicator the following mortality indicators were defined: in-acute orthopedic ward mortality, overall in-hospital mortality, in-hospital mortality before surgical intervention, overall operative-theatre mortality, in-hospital mortality after surgical intervention, 30-day mortality. Next to mortality other secondary outcome measures were defined: a) Readmission: 30-days readmission, 6-month readmission, b) Length of stay: interval time between admission and surgery (anesthetic induction), overall operative-theatre time, length of stay in the orthopedic ward, overall in-hospital length of stay; c) In-hospital pain score at 24 and 48 h postoperatively and at discharge d) Postoperative complications during hospitalization, e) Functional status at discharge, f) Mobility status at discharge, g) Proportion of patients returning to previous home status after discharge, h) Health Related Quality of Life (HRQL).

As one of the goals of care pathways is to enhance the compliance to evidence based components of treatment, next to outcome measures a set of process indicators were defined. We used the same methodology as for the outcome indicators by screening the international guidelines and performing an international Delphi study. The process indicators are: a) Risk assessment of pressure sores/ulcers, b) Prevention of pressure sores/ulcers, c) Surgery within 24 h after admission, d) Performance of X-ray pre-operatively, e) Pré-operative care and assessment: ensure general analgesia is adequate, f) Pré-operative care and assessment: assessment of anemia, g) Antithrombotic prophylaxis, h) Antibiotic prophylaxis, i) Assessment of cognitive status: at admission, postoperatively at start of mobilization, j) Assessment of nutritional status, k) Management of nutritional status, l) Mobilization within 24 h postoperatively, m) Geriatric Assessment pré-operatively, n) osteoporosis screening and management and o) discharge management.

These indicators were translated during a consensus meeting in actual measurements by a multidisciplinary expert panel, consisting of a geriatrician, an orthopedic surgeon, a physiotherapist, a clinical nurse specialist, and researchers of the EQCP-study [12,29]. To further understand why pathways work, information on the context of the organization is important. Within the EQCP study, a set of both generic and PFF-specific context indicators and team structure indicators was developed based on literature review, an international Delphi study, and expert opinion [12,29]. The literature searches and the international Delphi studies on both the outcome and process measures will be submitted as separate international publications as part of the EQCP research output. The details on each of the indicators (definition, numerator, denominator, inclusion & exclusion criteria) will be downloadable from the website of the European Pathway Association (http://www.E-P-A.org) as soon as the data of the study will be collected. In this way these indicators can be used by other teams who are in search for analyzing and optimizing their processes of care.

### Registration and ethical approval

The study has been registered as a cRCT at clinicaltrials.gov (identifier: NCT00962910). The ethical approval will be country-specific, but overall ethical approval will be obtained on three levels: (i) Ethical approval by the ethical committee of the coordinating centre on a country level; (ii) Ethical approval with regard to the participation in the intervention on a cluster level, i.e. by the ethical committee of each of the participating hospitals. These committees can agree or disagree with the overall approval of the coordinating centre. As indicated by the Medical Research Council, patient’s consent to participate in the study is not possible, because randomization occurred at the hospital level and not on the patient level. Moreover, the aim of the study is to improve adherence to evidence-based care through care pathways in the intervention group. In the control group, no intervention will be implemented and patients will only receive usual care. Participating in the intervention or control group should not imply any risk for the patients included; and (iii) Individual informed consent will be obtained from the patient with regard to the access of his/her record and participation in surveys. Approval of the ethical committee of the coordinating centre at Leuven University has already been obtained (identifier: ML5618/B32220096038) while approval in Portugal, Italy and Ireland is pending.

## Discussion

The EQCP study is the first international cluster-randomized controlled trial on the effect of care pathways in two specific patient groups: chronic obstructive pulmonary disease and proximal femur fractures, respectively. Three active components define the complex intervention: feedback on the actual performance, information on the evidence-based key interventions, and the design and implementation process. A cRCT design is combined with a realistic evaluation approach [26]. This allows analysis of the differences between the intervention and control groups in process and outcome indicators and evaluation of the context and implementation process in the intervention group [43]. As suggested by Berwick in 2008, it is not only important to understand if an intervention works but also why and under what circumstances it works [21]. The approach in the EQCP study will allow us to analyze if pathways work but also provide information on the when and how [29]. Hawe and colleagues argued that the crucial point in evaluating complex interventions lies in what is standardized. In complex interventions, standardization should not only apply to the components but also to the function and process of the intervention [44]. This is particularly important in pathway research as previously described by Panella et al. [28]. Rather than defining the components of the intervention as standard, what should be defined as standard are the steps in the change process [44]. In pathway research, the implementation process—a quality improvement cycle—that is executed is part of the intervention and that is why the improvement and implementation process is included as one of the basic active ingredients. One challenge in multicenter trials on care pathways—as well as in quality improvement methods in general—is to understand the context. Pawson and Tilley defined an action as causal only if its outcome is triggered by a mechanism acting in a specific context (context + mechanism = outcome) [26]. They argued that programs work (i.e., have good outcomes) to the extent that they introduce appropriate ideas and opportunities (the mechanism) to groups in the appropriate social and cultural conditions (the context) [21,26]. This realistic evaluation paradigm has already been used in pathway research [12,15,45] and was recently promoted by Berwick [21]. In pathways, the mechanism will need to be based on the basic active components as described above but the fine-tuning of the intervention will be based on the actual performance challenges and on the context of the organization and team involved. To fully understand what is happening while developing and implementing a care pathway, a set of team indicators and organizational factors will be measured and qualitative approaches will be used [23]. Process and outcome indicators will provide data to understand if pathways work, but the team indicators will be of help in understanding why and how they work. The design of this part of the study is currently being prepared and will be published later.

One important measure to analyze in this study will be the variation and impact of the length of stay on the outcomes, as this could lead to a difference in dose of the intervention. To analyze this issue and based on the discussions within the international research team and with the coordinators and clinicians in each of the participating countries, we included a set of measures on the length of stay and the process-flow. For each of the patients data are collected on the time a patient arrives in the hospital, the timing of the operation, the postoperative activities and the time of discharge. In this way we will be able to not only analyze the relation between the pre-operative and overall length of stay and the predefined process and outcome indicators, but we will also be able to analyze the variation within each of the sites, and between the sites in each country using a multilevel statistical approach. Additionally these data will provide information on the variation of the processes of care in these four European countries.

With this study, the European Pathway Association will be able to help health professionals and hospital managers in actively improving their quality and efficiency of care [29]. All findings will be reported as outlined in the CONSORT statement [46]. The teams will receive support in re-organizing the PFF-care processes and potentially use this implementation knowledge in other care processes. Teams will receive feedback on their actual performance including benchmark data compared to other international teams. As a result, an international network of high-performing teams on PFF will be built, making the EQCP project both a research study and a quality improvement project.

## Abbreviations

ARESS, Agenzia per i servizi sanitari regionali; CP, Care pathway; cRCT, Cluster randomized controlled trial; E-P-A, European Pathway Association; EQCP, European Quality of Care Pathway; ICC, Intracluster Correlation Coefficient; IFF, Inflation Factor; PDSA, Plan do study act; PFF, Proximal Femur Fracture.

## Competing interest

The authors declare that they have no competing interests.

## Authors’ contribution

KV, WS, CL, SD, SB, MP contributed to the draft and final version of the paper. JP, FL, PB and RM have been involved in the setup of the study and contributed to the final version of the paper. JP is chairman of the steering committee of the EQCP research group. KV, SB, CL, FL, SD and MP supervised the selection of the main outcome indicators and the clinical content of the intervention. MP, KV and WS have the scientific lead of the study. KV is international coordinator of the study. All members of the EQCP Study Group have been involved in the organization of the study in the four participating countries. All authors have read and approved the final manuscript.

## Pre-publication history

The pre-publication history for this paper can be accessed here:

http://www.biomedcentral.com/1472-6963/12/124/prepub
